# hicap: *In Silico* Serotyping of the Haemophilus influenzae Capsule Locus

**DOI:** 10.1128/JCM.00190-19

**Published:** 2019-05-24

**Authors:** Stephen C. Watts, Kathryn E. Holt

**Affiliations:** a Department of Biochemistry and Molecular Biology, Bio21 Molecular Science and Biotechnology Institute, University of Melbourne, Melbourne, Victoria, Australia; b Department of Infectious Diseases, Central Clinical School, Monash University, Melbourne, Victoria, Australia; c London School of Hygiene & Tropical Medicine, London, United Kingdom; University Hospital Münster

**Keywords:** *Haemophilus influenzae*, capsule, genomics, serotyping, surveillance

## Abstract

Haemophilus influenzae exclusively colonizes the human nasopharynx and can cause a variety of respiratory infections as well as invasive diseases, including meningitis and sepsis. A key virulence determinant of H. influenzae is the polysaccharide capsule, of which six serotypes are known, each encoded by a distinct variation of the capsule biosynthesis locus (*cap*-a to *cap*-f). H. influenzae type b (Hib) was historically responsible for the majority of invasive H. influenzae disease, and its prevalence has been markedly reduced in countries that have implemented vaccination programs targeting this serotype. In the postvaccine era, nontypeable H. influenzae emerged as the most dominant group causing disease, but in recent years a resurgence of encapsulated H. influenzae strains has also been observed, most notably serotype a. Given the increasing incidence of encapsulated strains and the high frequency of Hib in countries without vaccination programs, there is growing interest in genomic epidemiology of H. influenzae. Here we present hicap, a software tool for rapid *in silico* serotype prediction from H. influenzae genome sequences. hicap is written using Python3 and is freely available at https://github.com/scwatts/hicap under the GNU General Public License v3 (GPL3). To demonstrate the utility of hicap, we used it to investigate the *cap* locus diversity and distribution in 691 high-quality H. influenzae genomes from GenBank. These analyses identified *cap* loci in 95 genomes and confirmed the general association of each serotype with a unique clonal lineage, and they also identified occasional recombination between lineages that gave rise to hybrid *cap* loci (2% of encapsulated strains).

## Abstract (precis)

Haemophilus influenzae exclusively colonizes the human nasopharynx and can cause a variety of respiratory infections as well as invasive diseases, including meningitis and sepsis. A key virulence determinant of H. influenzae is the polysaccharide capsule, of which six serotypes are known, each encoded by a distinct variation of the capsule biosynthesis locus (*cap*-a to *cap*-f).

## INTRODUCTION

Haemophilus influenzae is a pleomorphic Gram-negative bacterium that is exclusive to humans, typically colonizing the upper respiratory tract and occasionally causing disease. It was the first free living organism to be completely sequenced and served as a stepping stone toward DNA sequencing technology development in preparation for the Human Genome Project (1). H. influenzae is often classified on the basis of the production and antigenicity of polysaccharide capsule. Strains that produce capsule are divided into six serotypes (H. influenzae a to f [Hia to Hif]), and nonencapsulated strains are designated nontypeable H. influenzae (NTHi) (2).

**FIG 1:**
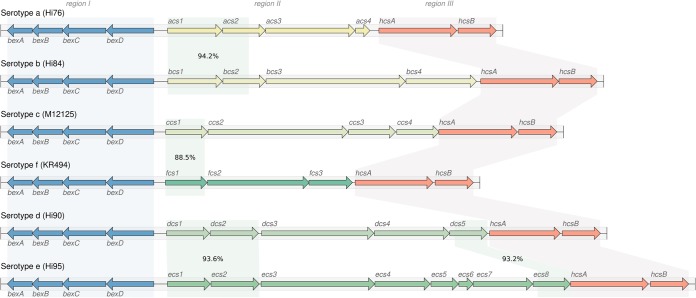
Schematic representation of the six known H. influenzae *cap* loci. Capsule nucleotide sequences and annotations were collected from genome assemblies representing each of the six serotypes. Shading indicates homologous regions between reference loci as determined by BLAST identity values shown in region II. Regions I and III are homologous across the entire sequence for all loci, with nucleotide identities of ≥87% and ≥90%, respectively.

Biosynthesis of the polysaccharide capsule is controlled by the *cap* loci (*cap*-a to *cap*-f), each of which includes three contiguous but functionally distinct regions (I, II, and III) (Fig. 1). Regions I and III are common to all six *cap* loci and are associated with cellular transport (*bex* operon, region I) and posttranslational processing (*hcs* operon, region III) (3, 4). Region II encodes several genes involved in polysaccharide biosynthesis that are specific to each serotype (Fig. 1) (5–9). The *cap* locus is regularly subject to duplication, deletion, and interruption (10, 11). For example the *cap*-b locus is often duplicated, creating two tandem copies of the locus flanked by IS*1016*, and regularly coincides with a 1.2-kbp deletion of the terminal *bexA*-IS*1016* copy (9). The arrangement and copy number of *cap* locus genes also have clinical relevance, as certain structural variants are associated with increased levels of virulence (12).

H. influenzae is capable of causing a variety of respiratory infections and invasive diseases. Prior to the introduction of capsular conjugate vaccines against Hib in the 1980s, this serotype was responsible for almost all H. influenzae-related morbidity and mortality (13). In the period subsequent to wide-spread adoption of childhood Hib vaccination programs, the incidence of Hib-related disease reduced markedly (14). However, following implementation of Hib vaccination programs, disease caused by NTHi has been increasing globally. The prevalence of disease caused by other encapsulated strains is also increasing at an alarming rate, and the Hia disease burden now exceeds that of Hib during the pre-Hib vaccination era in some regions and populations (15). A recent report of particular interest found that Hia constituted 50% of all H. influenzae cases between 2010 and 2015 in northwestern Ontario, Canada (16). Importantly Hib also remains an issue in countries that have not implemented a vaccination program (17).

Public health and clinical laboratories are now beginning to incorporate whole-genome sequencing (WGS) technologies into diagnostic, outbreak, and surveillance programs (18, 19). The departure from molecularly based diagnostics has been driven largely by the considerably higher resolution and accuracy afforded by WGS (20). Currently there are no dedicated tools for H. influenzae serotype prediction that seek to leverage WGS data for *cap* locus detection. The need for such a tool continues to grow with the resurgence of encapsulated H. influenzae and the increasingly routine use of WGS in the public health setting.

Here we describe hicap, a software tool specifically designed for rapid *in silico* serotype prediction from H. influenzae WGS data. hicap is an open source Python3 package and is freely available at https://github.com/scwatts/hicap under a GNU General Public License v3 (GPLv3). We further apply hicap to identify and extract *cap* loci from all H. influenzae genomes currently available in GenBank, and we explore the diversity and distribution of these loci in the H. influenzae population.

## MATERIALS AND METHODS

### hicap implementation and validation.

**FIG 2:**
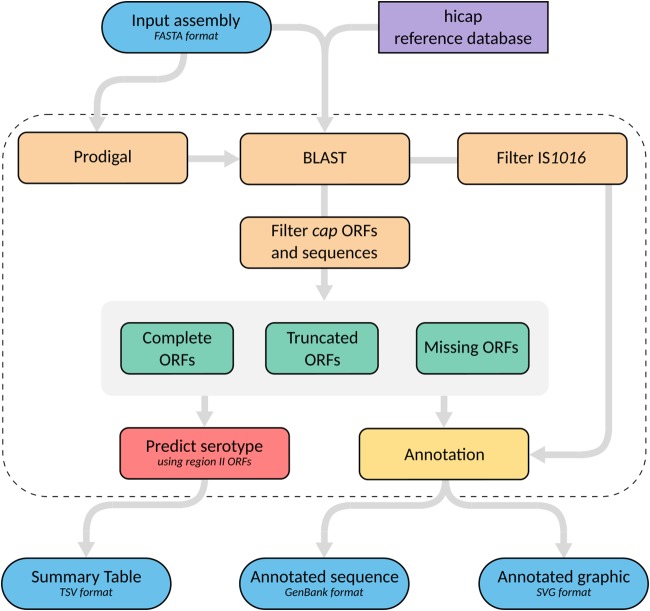
Summary of the hicap serotype prediction method. hicap takes an assembled genome in FASTA format as input and detects all open reading frames (ORFs) using Prodigal. Constituent *cap* genes and IS*1016* copies are identified by performing alignments of either the ORF sequence or input assembly sequence against the reference database using BLAST+. The identified *cap* genes and IS*1016* alignments are then used to inform structural composition of the locus. Serotype is predicted using the gene complement information of region II.

hicap uses a reference database to identify genes expected in the six *cap* loci (*cap*-a to *cap*-f). To this end, a curated nucleotide sequence database of *cap* locus genes was constructed by extracting the protein-coding sequences annotated from *cap* loci in publicly available sequences of well-defined H. influenzae serotypes (Table 1). The process adopted by hicap to perform serotype prediction from WGS assemblies by using this database is described in Fig. 2

**TABLE 1:** *cap* locus sequences used to create the hicap reference database

Gene or region	Strain	Accession no.	Reference
*bexA*	KR494	GCA_000465255.1	43
*bexB*	KR494	GCA_000465255.1	43
*bexC*	KR494	GCA_000465255.1	43
*bexD*	KR494	GCA_000465255.1	43
*cap*-a	Hi76	ERX1834399	44
*cap*-b	NCTC 8468	ERX704106	45
*cap*-c	Hi85	ERX1834408	44
*cap*-d	ATCC 9008 (*cap* locus)	HQ424464.1	Haemophilus influenzae ATCC 9008
*cap*-e	hi467	GCA_001975845.1	46
*cap*-f	KR494	GCA_000465255.1	43
*hcsA*	KR494	GCA_000465255.1	43
*hcsB*	KR494	GCA_000465255.1	43
IS*1016*	Hae18	X59756.1	47

First, all open reading frames (ORFs) are identified in the query assembly using Prodigal (21). Each ORF nucleotide sequence then is queried against the hicap reference database using BLAST+ (22). The resulting alignments are filtered on the basis of subject coverage and nucleotide identity. The default parameters to designate an ORF a complete match to a *cap* locus gene are subject coverage of ≥80% and nucleotide identity of ≥70%. Often *cap* genes that are expected to be present lack a complete match to any ORF annotated by Prodigal. This typically occurs when an ORF in the Prodigal annotation has been truncated due to missense mutations, mobile elements, or incomplete assembly. hicap infers the number of genes missing from the predicted *cap* locus by examining the count of complete ORFs and comparing this to the expected count for the complete form of that locus.

Generally, hicap will attempt to find at least one copy of each gene expected in the *cap* locus. In the case that there are missing genes, hicap searches the remaining ORF database alignments for the expected gene fragment(s) using more relaxed filtering (defaults for this are alignment length of ≥60 bp and nucleotide identity of ≥80%). Failing this, hicap employs BLAST+ to identify regions of the input assembly that are homologous to missing genes proximal to the predicted *cap* locus (filtering alignments with a bit score of ≤200). An ORF or sequence is designated truncated if it is identified by either of these adjusted filters but does not meet the criteria for a complete match. Additionally, hicap searches for IS*1016* in the *cap* locus and nearby regions by aligning the reference IS*1016* sequence with the input assembly using BLAST+.

**FIG 3:**
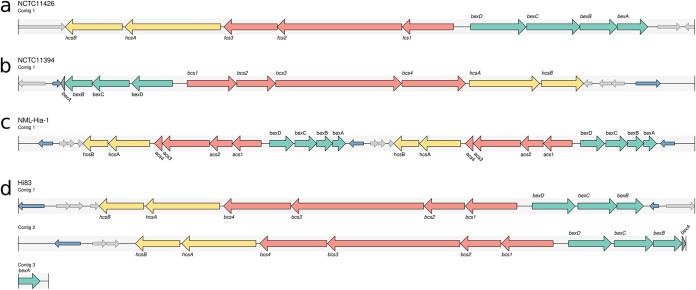
Examples of hicap visualization for selected genomes. *cap* locus genes are annotated as large arrows with the direction representing the strand. Genes of the *cap* locus are colored to indicate region (region I, green; region II, red; region III, yellow). A truncated *cap* gene is given a darker shade of color for the respective region. Copies of IS*1016* are denoted as small blue arrows, and open reading frames that do not generally belong to the *cap* locus are show as small gray arrows. (a) The complete and contiguous annotation of the NCTC 11426 *cap*-f locus. (b) The NCTC 11394 *cap*-b locus, which contains a truncated *bexA* gene and two copies of IS*1016*. (c) A duplication of the *cap*-a locus is observed in the assembly of NML-Hia-1. (d) The *cap*-b locus of Hi83 is also duplicated but is present across multiple contigs in the input assembly, as represented by multiple tracks.

The resulting set of alignments and ORFs are used to predict serotype and various locus characteristics. Specifically, hicap predicts serotype by considering all complete and truncated alignments of region II genes. The predicted serotype is defined as the serotype observed to have the most complete set of region II genes. Where an ORF has multiple alignments to the hicap database, a single best alignment is selected on the basis of E value, with ties broken by bit score. ORFs identified as belonging to the *cap* locus and surrounding region are summarized in a tab-delimited report file, and the annotated *cap* locus sequence is output in GenBank format. A visualization of the locus annotation is also created using the graphics module in Biopython (23) and output in SVG format (examples are shown in Fig. 3).

To test the ability of hicap to predict serotypes, we reviewed the literature and identified publicly available WGS data for H. influenzae isolates with known serotypes (Table 2). The genome assemblies were downloaded and analyzed using hicap run with default parameters. For 26 isolates, only read data were available; hence, *de novo* assemblies were generated using SPAdes v3.12.0 (24) prior to analysis with hicap. All validation was performed using hicap v1.0.0. The full set of assemblies used for testing is available in FigShare (https://doi.org/10.26180/5c352c5110712). When discrepancies were observed between expected the serotype and hicap results, the output of nucleotide BLAST+ v2.7.1+ searches of genome assemblies against the hicap database was manually inspected.

**TABLE 2:** Strains used in the validation of the hicap method, with predicted serotype and fragmentation status of the *cap* locus as determined by hicap

Strain	Serotype		Fragmented locus	Assembly identifier^*a*^	Read identifier
	Described	Predicted by hicap			
Hi75	a	a	No		ERX1834398
Hi76	a	a	No		ERX1834399
Hi77	a	a	No		ERX1834400
Hi78	a	a	No		ERX1834401
Hi79	a	a	No		ERX1834402
Hi609	a	a	No	GCA_003363335.1	
Hi642	a	a	No	GCA_003363355.1	
NML-Hia-1	a	a	No	GCA_001856725.1	
10810	b	b	No	GCA_000210875.1	
ATCC 10211	b	b	Yes	GCA_001997355.1	
Hi80	b	b	No		ERX1834403
Hi81	b	b	Yes		ERX1834404
Hi82	b	b	Yes		ERX1834405
Hi83	b	b	Yes		ERX1834406
Hi84	b	b	No		ERX1834407
NCTC 13377	b	b	No	GCA_900478275.1	
NCTC 8468	b	b	No	NCTC 8468 (Sanger FTP)	
Hi85	c	c	No		ERX1834408
Hi86	c	c	No		ERX1834409
Hi87	c	c	No		ERX1834410
Hi88	c	c	No		ERX1834411
M12125	c	c	No	GCA_003351605.1	
M17648	c	c	No	GCA_003351465.1	
Hi89	d	d	No		ERX1834412
Hi90	d	d	No		ERX1834413
hi467	e	e	No	GCA_001975845.1	
Hi91	e	e	Yes		ERX1834414
Hi92	e	e	No		ERX1834415
Hi93	e	e	No		ERX1834416
Hi94	e	e	No		ERX1834417
Hi95	e	e	No		ERX1834418
NCTC 8455	e	e	No	GCA_900478735.1	
Hi100	f	f	No		ERX1834168
Hi96	f	f	No		ERX1834419
Hi97	f	f	No		ERX1834420
Hi98	f	f	Yes		ERX1834421
Hi99	f	f	No		ERX1834422
KR494	f	f	No	GCA_000465255.1	
NCTC 11394	f	b	No	NCTC 11394 (Sanger FTP)	
NCTC 11426	f	f	No	GCA_900475755.1	
WAPHL1	f	f	Yes	GCA_002237715.1	
86-028NP	NTHi	No *cap* locus		GCA_000012185.1	
PittEE	NTHi	No *cap* locus		GCA_000016465.1	
Rd KW20	NTHi	No *cap* locus		GCA_000027305.1	

a Assemblies that were available for each isolate were downloaded and screened. Assemblies obtained from the Sanger FTP (https://sanger.ac.uk/resources/downloads/bacteria/nctc) were additionally converted from GFF3 to FASTA format. Where an assembly was not available for an isolate, read sets were downloaded and assembled using SPAdes (as described in Materials and Methods) before screening. All assemblies used for testing are available through FigShare (https://doi.org/10.26180/5c352c5110712).

### *cap* locus distribution, variation, and recombination.

To demonstrate a practical application of hicap, we investigated the distribution of capsular serotypes predicted by hicap among all H. influenzae genomes available in NCBI GenBank as of 8 October 2018 (*n* = 698, listed in Table S1 in the supplemental material). Whole-genome assemblies were downloaded via FTP, and a phylogeny was constructed using mashtree v0.33 (https://github.com/lskatz/mashtree) (25). Genomes were excluded from analysis where the assembly length was more than four standard deviations from the mean or the genomic content was sufficiently dissimilar to that of H. influenzae (*n* = 7). Specifically genomic content was assessed by simulating 50,000 error-free reads using wgsim v0.3.1-r13 (https://github.com/lh3/wgsim), which were taxonomically classified by centrifuge v1.0.4-beta (26) and samples with ≤80% H. influenzae reads excluded.

The sequence type (ST) for each assembly was determined via comparison to the multilocus sequence typing (MLST) database for H. influenzae (https://pubmlst.org/hinfluenzae) (27) using mlst v2.15 (https://github.com/tseemann/mlst). Capsular serotypes were inferred using hicap v1.0.0 with the default settings. Nucleotide sequence homology between hybrid *cap* loci was assessed by BLAST+ v2.7.1+ and visualized using genoPlotR v0.8.7 (28) in R v3.4.4 (29). The mashtree phylogeny was annotated with the ST and predicted capsular serotype in R v3.4.4 using ggtree v1.12.7 (30).

To establish the relationship between capsular serotype and allelic variants of genes encoded in regions I and III, we constructed individual gene trees. Nucleotide sequences were extracted for all complete region I and III genes that were detected by hicap during analysis of the H. influenzae GenBank data set. For each individual gene, nucleotide sequences were aligned using MAFFT v7.407 with default settings (31) and phylogenies inferred from the alignment using FastTree v2.1.10 with the general time-reversible substitution model (32). Nucleotide divergence was calculated using ape v5.2 (33) in R v3.4.4 from gene nucleotide alignments.

## RESULTS AND DISCUSSION

### hicap validation.

To validate hicap as a tool for *in silico* serotyping, we analyzed 41 publicly available H. influenzae genomes with reported serologically confirmed capsule types, including representatives for each of the six serotypes and three NTHi strains (Table 2). The results show that hicap robustly identifies the H. influenzae *cap* locus even in highly discontiguous assemblies. For each *cap* locus, the completeness, presence of truncated genes, duplication, contiguity, and serotype were correctly reported.

Capsule loci were detected by hicap in 41/41 genomes with reported serotypes and in 0/3 serologically determined NTHi genomes (Table 2). The predicted serotype matched the reported serotype in 40/41 cases (98%) (Table 2). We found that hicap yielded accurate predictions even from draft genomes where the *cap* locus was fragmented across multiple contigs (observed in 7 genomes from the validation set). Examples of the *cap* loci identified and visualized using hicap are shown in Fig. 3.

The single genome with a discrepancy between predicted and reported serotype, NCTC 11394, is described as Hif in the National Collection of Type Cultures (NCTC) but was confidently assigned Hib by hicap analysis of the completed PacBio genome assembly (Fig. 3b). Manual assessment of the *cap* locus in the NCTC 11394 genome assembly additionally confirmed the presence of a complete *cap*-b locus (uninterrupted, in a single contig) and the absence of any *cap*-f region II genes, with all expected *cap*-b protein-coding genes present at ≥95% coverage and ≥84% homology to those annotated in the *cap*-b reference sequence (excluding a truncated *bexA* gene). The standard slide agglutination test classically used for serological typing of H. influenzae has been shown to lack specificity and has been estimated to yield incorrect results at a rate of 17.5% (34). It therefore appears likely this discordance is due to inaccuracies in the described serotype rather than misidentification by hicap.

### *cap* locus distribution and variation.

To demonstrate the utility of *in silico* serotype prediction with hicap, we used it to investigate all publicly available H. influenzae genomes in GenBank that passed quality filtering criteria (*n* = 691; see Table S1 in the supplemental material). hicap identified a complete *cap* locus in 95/691 (13.7%) genomes (8 *cap*-a, 54 *cap*-b, 4 *cap*-c, 1 *cap*-d, 20 *cap*-e, and 8 *cap*-f). All genomes contained either zero or one *cap* locus type, but duplication events were observed in 15/95 (15.8%) *cap*-positive genomes (14 *cap*-b and 1 *cap*-a).

Duplication of the *cap*-b locus has been frequently reported and is associated with enhanced virulence, conferred by an increased ability to produce capsule (12, 35). This duplication is thought to be driven by copies of IS*1016* flanking the capsule locus in some isolates. A common variant of the duplicated *cap*-b locus involves the deletion of 1.2 kbp in one copy of region I, resulting in the truncation of *bexA* and IS*1016*. We observed this duplication deletion variant in 14/54 (26.0%) predicted Hib genomes. Complete *cap*-b duplication without truncation of *bexA* was not observed. In addition, hicap identified a single isolate (NML-Hia-1) containing a tandem duplication of the *cap*-a locus. Strains identified to be carrying *cap* loci were not assessed for capsule production; however, several of these strains are known to synthesize capsule (e.g., 10810 and NML-Hia-1).

**FIG 4:**
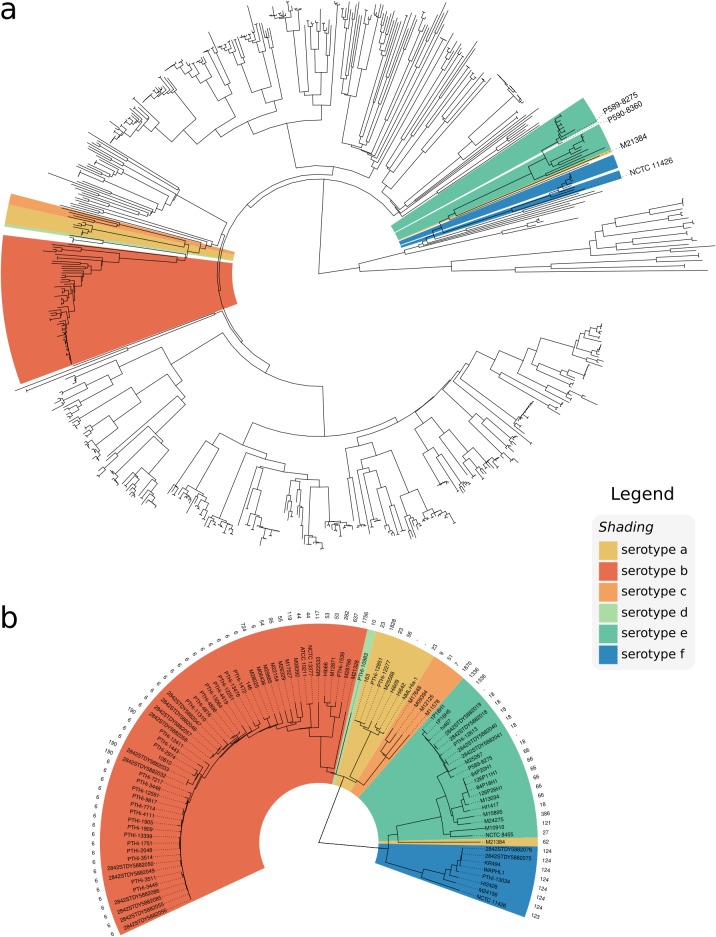
Whole-genome neighbor-joining phylogeny inferred from MASH distances of assemblies in the GenBank data set. Isolates are annotated with the respective serotype as predicted by hicap. (a) Distribution of capsular serotypes in the complete data set. (b) The phylogeny subtree including only isolates that contained a *cap* locus, additionally annotated with the sequence type.

To examine the distribution of *cap* loci in the H. influenzae population, we constructed a whole-genome phylogeny (Fig. 4) and inferred STs according to the H. influenzae MLST scheme (Table 3). We observed a high degree of exclusivity for STs in regard to predicted capsular serotypes, with each ST containing zero or one *cap* locus serotype.

**TABLE 3:** Sequence types associated with each serotype in the GenBank data set

Serotype	ST	No.	Frequency (%) of serotype within ST
a	23	3	100
		2	
	1828	1	100
	56	1	100
	62	1	100
b	6	38	100
	190	3	100
	44	2	100
	53	2	100
	117	1	100
	119	1	100
	1756	1	100
	282	1	100
	54	1	100
	55	1	100
	637	1	100
	724	1	100
	95	1	100
c	1870	1	100
	51	1	100
	7	1	100
	9	1	100
d	10	1	100
e	18	7	87.5
	66	6	100
		2	
	1336	2	100
	121	1	100
	27	1	100
	386	1	100
f	124	7	87.5
	123	1	100

The whole-genome phylogeny confirmed that encapsulated strains are relatively clonal and are generally restricted to serotype-specific monophyletic clades (Fig. 4a), suggesting that each serotype emerged once within the H. influenzae population. This is consistent with earlier studies based on electrophoretic typing (36, 37), 16S rRNA (38), MLST (27), and WGS (39). Here, the whole-genome phylogeny resolves the monophyletic nature of each capsule locus with respect to phylogenetic lineage on a larger scale and in greater detail.

hicap did not detect *cap* loci in a small number of isolates within these serotype-specific clades, indicating occasional capsule loss. For example, P590-8360 clustered with the Hie clade (Fig. 4), but no *cap* locus was identified by hicap or by manual inspection of the assembly data. The high nucleotide identity between P590-8360 and the *cap*-e-positive strain P589-8275 along the rest of the genome (Fig. 4a) suggests that loss of the *cap*-e locus in the P590-8360 genome is the mostly likely explanation. Indeed, the loss of capacity to synthesize capsule has previously been observed to occur by partial or complete deletion of the *cap* locus (39), and the rate of spontaneous capsule loss is estimated to occur at a frequency of 0.1 to 0.3% (40). Our data are consistent with deletion of the *cap* locus being a cause of this phenomenon. Interestingly one *cap*-a genome (M21384) falls within the *cap*-e serotype-specific clade, suggesting possible recombination in this strain (Fig. 4) (further evidence for this is discussed below).

**FIG 5:**
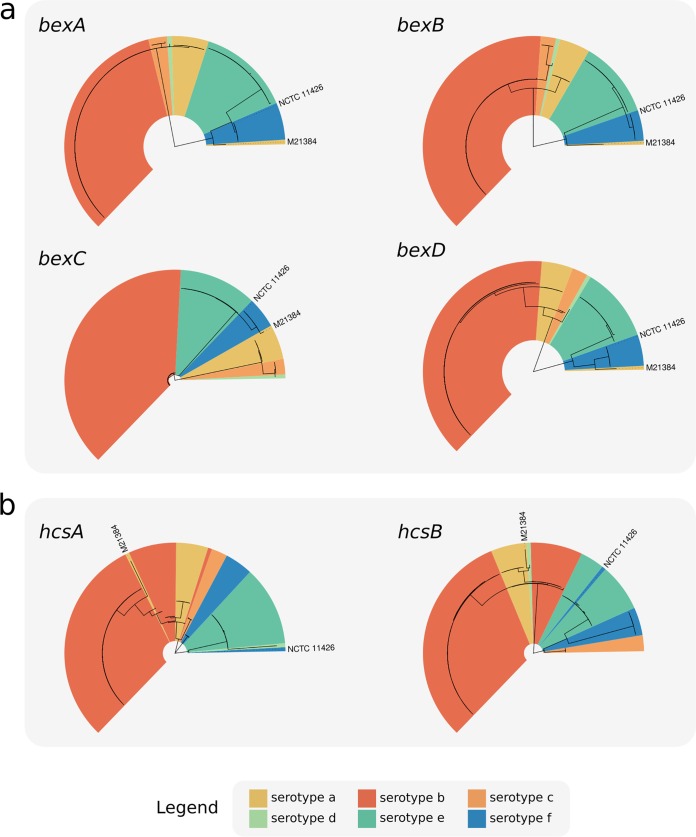
Phylogenies of all complete *cap* locus region I (a) and III (b) genes identified in the GenBank data set. FastTree was used to recover phylogenies from MAFFT gene nucleotide sequence alignments, and isolates were annotated using the serotype as predicted by hicap.

The serotype-specific clades cluster into two superclades within the H. influenzae phylogeny: one containing *cap* loci of Hia, Hib, Hic, and Hid and the other containing Hie and Hif *cap* loci (Fig. 4). Individual gene trees for the region I (*bex*) and III (*hcs*) genes show the same two-clade structure (Fig. 5) as the core genomes of their host strains. This observation is consistent with diversification of these *cap* locus regions *in situ* within their host chromosomes following introduction into two distinct H. influenzae superclade ancestors. While there is a general lack of homology between region II genes (Fig. 1), two of the three pairs that do show a measure of homology (*cap*-c/*cap*-f and *cap*-d/*cap*-e) span both superclades; hence, the evolutionary history of region II (and thus the distinct capsular serotypes) remains cryptic.

Variation in each region I or III gene was associated with serotype, suggesting that the sequence of any could potentially be used to predict capsule type with a relatively high degree of certainty (Fig. 5). Indeed, both *bexA* and *bexB* have been proposed and used in single-gene PCR assays for the purpose of serotyping (41, 42). Here the gene *bexB* showed the greatest differentiation between serotype-specific alleles (0.63% to 17.71% median pairwise nucleotide divergence; see Fig. S1 in the supplemental material) and contained only serotype-specific monophyletic clades in the gene tree. These data suggest *bexB* to be the most suitable single marker gene for use in PCR or sequenced-based prediction of serotype. In contrast, *bexA* showed less differentiation then *bexB*, particularly between serotypes a, b, c, and d (0.17% to 0.85% median pairwise nucleotide divergence).

The exceptions to the general association between region I/III genes and predicted serotype were two isolates, M21384 and NCTC 11426 (labeled in Fig. 5). hicap predicted isolates M21384 and NCTC 11426 to be of serotype a and serotype f, respectively. However, both carry *cap* region I and/or III gene sequences distinct from other strains sharing the same *cap* II region type (and thus the same predicted serotype), indicative of recombination involving the *cap* locus within these isolates. Thus, there is evidence for occasional recombination within the *cap* locus between the different serotype-specific variants, which would limit the accuracy of any single marker gene-based approach to serotype prediction.

### Recombination affecting the *cap* locus.

The isolate M21384 was the only exception to clonal clustering by serotype in the whole-genome phylogeny (Fig. 4). While this isolate is predicted to be Hia based on the presence of *cap*-a region II genes, the genome falls outside the Hia clade and within the Hie/Hif superclade (Fig. 4b). In all gene trees, M21384 also did not cluster in the expected Hia serotype clade, suggesting that there has been recombination within the *cap* locus of this isolate (see Fig. 5). Similarly in the *hcsA* and *hcsB* gene trees, the isolate NCTC 11426 did not cluster with the expected serotype Hif clade (Fig. 5b). Given the phylogenetic relation of M21384 and NCTC 11426 to other capsular serotypes, it was suspected that these two strains result from recombination events affecting the *cap* locus (representing a 2% recombination rate involving the *cap* locus).

**FIG 6:**
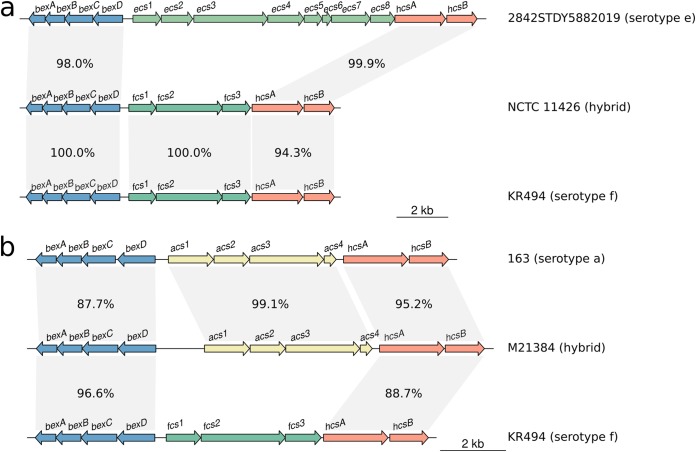
Homology plots generated using R and genoPlotR, showing the *cap* loci of two isolates which appear to have been subject to recombination. Different regions of the NCTC 11426 (a) and M21384 (b) *cap* loci show varying homology to different reference *cap* loci, suggesting a recombinogenic ancestry.

To better understand the recombinant *cap* loci in isolates M21384 and NCTC 11426, we first examined their positions in the whole-genome phylogeny (Fig. 4) and the *cap* locus gene trees (Fig. 5) and then compared the full-length *cap* locus sequences of both isolates to reference *cap* locus sequences (Fig. 6). NCTC 11426 (predicted to be Hif) belongs to the Hif clade in the whole-genome tree and carries typical *cap*-f regions I and II but contains region III genes more similar to those from *cap*-e (Fig. 6a). Hence, it appears that the *cap* locus of NCTC 11426 has resulted from a small recombination event between a Hif clade strain and the *cap* locus from a Hie clade strain. M21384 clusters within the Hie/Hif superclade of the whole-genome phylogeny and carries *cap*-f-like region I genes (Fig. 5a and 6b). However, this isolate carries *cap*-a-like region II genes with a *cap*-b-like region III gene (*hcsA*) (Fig. 5b and 6b). The gene content of the M21384 *cap* locus suggests at least one recombination event involving import of foreign *cap* locus DNA into a Hif strain. It would be interesting to ascertain whether the isolates with recombinant *cap* loci described here do in fact express capsule and, if so, to then establish the serological phenotype. However, to our knowledge serotyping has not been performed for either strain, or the data are not publicly available.

### Conclusion.

The need for new tools and methods that leverage WGS continues to become increasingly pivotal with the adoption of WGS by public health laboratories. In this study, we validated and demonstrated the robustness of hicap for prediction of H. influenzae serotype and capsule locus structure. The application of hicap to WGS enables rapid and accurate acquisition of capsule information to aid genomic studies at both individual and population scales. We were also able to explore the diversity and distribution of *cap* loci in the H. influenzae population at unprecedented nucleotide resolution, identifying a likely misreported serotype in NCTC and describing two novel H. influenzae *cap* locus recombinants. The resurgence of disease caused by encapsulated H. influenzae and the potential for further antigenic diversification through recombination present a potential public health issue. An important question is whether the geographically disparate reports of increasing cases of infection with non-Hib encapsulated strains reflect the emergence and wide dissemination of a small number of highly successful disease-causing subclones (i.e., a rare but worrying event) or multiple independent events reflecting sporadic but localized outbreaks of non-Hib disease. hicap will facilitate extracting answers to these and other questions from genomic surveillance data.

## Supplementary Material

Supplemental file 1

Supplemental file 2
