# Supplementary material for: hicap: In Silico Serotyping of the Haemophilus influenzae Capsule Locus
Source: J Clin Microbiol. 2019 May 24;57(6):e00190-19. doi: 10.1128/JCM.00190-19 (PMC6535587; doi:10.1128/JCM.00190-19)
Supplement: Supplemental file 1 [file JCM.00190-19-s0001.pdf]

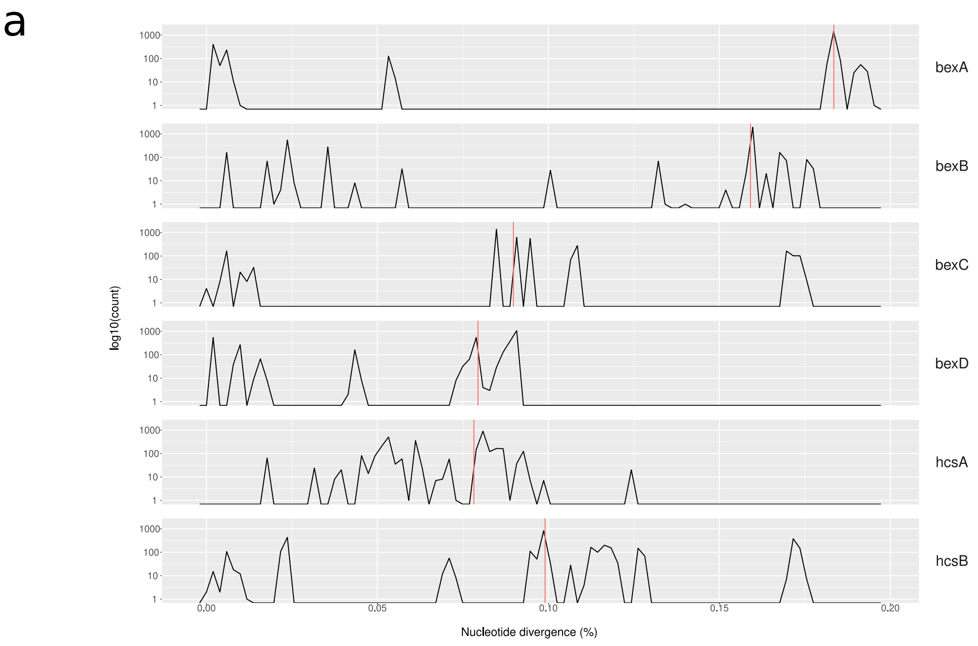

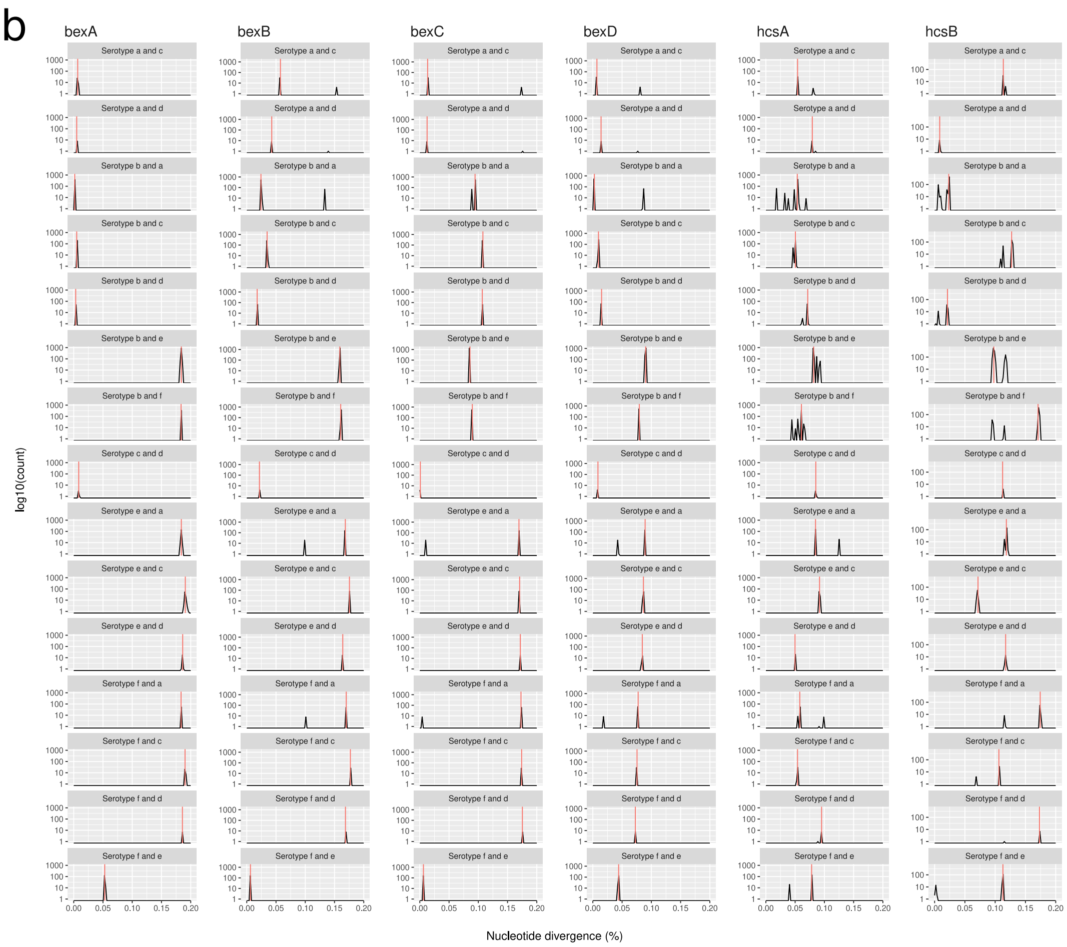

FIG S1. Distributions of nucleotide divergence measured for every gene sequence in different serotype groups from the GenBank dataset with median divergence indicated by red lines. Nucleotide divergence calculated by ape::dist.dna from MAFFT alignments for all *cap* region I and III gene sequences. (a) Combined distributions for all pair-wise nucleotide divergence measures of each gene. (b) Distribution of pair-wise measurements for each sequence between serotype groups of each gene.
